# Durable complete remission of pulmonary metastases with bevacizumab plus olaparib maintenance therapy in homologous recombination–deficient gynecologic large-cell neuroendocrine carcinoma: A case report

**DOI:** 10.1016/j.gore.2026.102114

**Published:** 2026-05-19

**Authors:** Yasushi Iida, Yoko Saito, Miwako Shimazaki, Teppei Ichikawa, Taichi Irie, Jun Matsushima, Makoto Iizuka, Satoshi Takakura

**Affiliations:** a Department of Obstetrics and Gynecology, Dokkyo Medical University Saitama Medical Center, Koshigaya, Saitama 343-8555, Japan; b Department of Pathology, Dokkyo Medical University, Saitama Medical Center, Koshigaya, Saitama 343-8555, Japan

**Keywords:** Neuroendocrine carcinoma, Bevacizumab, Olaparib, Homologous recombination–deficient, Case report

## Abstract

Gynecologic neuroendocrine carcinoma (NEC) is a rare and aggressive malignancy with limited treatment options and poor prognosis. Homologous recombination deficiency (HRD) is an established predictive biomarker for poly(ADP-ribose) polymerase (PARP) inhibitor response in ovarian cancer, but its clinical relevance in NEC remains unclear.

Case presentation

A 71-year-old woman presented with a large pelvic mass and multiple pulmonary metastases. Primary debulking surgery was performed, and histopathology revealed large-cell NEC (LCNEC) of presumed gynecologic origin. Postoperatively, the patient received paclitaxel–carboplatin plus bevacizumab. HRD testing demonstrated a high genomic instability score without BRCA1/2 mutations. After six cycles of chemotherapy, the pulmonary lesions showed a partial response. Maintenance therapy with bevacizumab plus olaparib was initiated, and the patient achieved complete radiologic remission, remaining disease-free for 1 year.

Conclusion

HRD-positive gynecologic NEC may respond to PARP inhibitor–based therapy even in the absence of BRCA mutations. HRD assessment may help identify patients with NEC who could benefit from targeted treatment strategies.

## Highlights

• HRD-positive gynecologic LCNEC responded to PARP inhibitor therapy.
• Complete remission was achieved with bevacizumab plus olaparib.
• HRD may predict benefit beyond BRCA-mutated tumors.
• PARP inhibitors may be effective in selected cases of NEC.

## 1 Introduction

Neuroendocrine carcinoma (NEC) is a poorly differentiated, high-grade epithelial malignancy characterized by aggressive clinical behavior (Rindi et al., 2022). NEC most commonly arises in the lung and, according to the 2022 World Health Organization classification, is subdivided into small cell NEC (SCNEC) and large-cell NEC (LCNEC) (Rindi et al., 2022). SCNEC, regardless of its primary site, is typically characterized by biallelic inactivation of TP53 and RB1, resulting in genomic instability and uncontrolled proliferation. In contrast, LCNEC exhibits a more heterogeneous molecular profile with less consistent TP53 and RB1 alterations compared to SCNEC.

NEC of the gynecologic tract is rare, accounting for less than 1% of all cases (Dasari et al., 2018). Among the gynecologic organs, the uterine cervix is the most common primary site, followed by the ovary and, less frequently, the uterine corpus (Dasari et al., 2018). Gynecologic NECs typically demonstrate aggressive behavior with poor prognosis, owing to a higher frequency of lymphovascular invasion and a greater propensity for early distant metastasis and recurrence compared to non-neuroendocrine gynecologic cancers (Stumpo et al., 2023). Because of their rarity, optimal systemic treatment strategies have not been established, and management is largely extrapolated from treatment approaches for NECs from other organs, such as small cell lung cancer, or from more common gynecologic malignancies (Stumpo et al., 2023).

Some LCNECs share molecular features with conventional non-neuroendocrine carcinomas of the same anatomical site, suggesting that NECs may arise either from the transformation of epithelial carcinomas with acquired neuroendocrine differentiation or from neuroendocrine precursor cells (Rindi et al., 2022). Endometriosis is a well-recognized precursor lesion for several gynecologic malignancies, particularly ovarian clear cell and endometrioid carcinomas (Yachida et al., 2021). Carcinomas arising from endometriosis may occasionally undergo neuroendocrine differentiation resulting in NEC, and rare case of LCNEC arising from endometriosis have been reported (Yanagita et al., 2023).

Homologous recombination deficiency (HRD) is a genomic phenotype characterized by impaired DNA double-strand break repair and resultant genomic instability. In epithelial ovarian cancer, HRD is assessed using genomic scar-based assays (e.g., genomic instability score [GIS]) and has been established as a predictive biomarker for sensitivity to platinum agents and poly(ADP-ribose) polymerase (PARP) inhibitors, even in the absence of BRCA1/2 mutations (Ray-Coquard et al., 2019). In contrast, the prevalence and therapeutic relevance of HRD in gynecologic NEC remain poorly defined.

This report describes a case of advanced pelvic LCNEC with multiple pulmonary metastases, possibly arising from Müllerian-derived tissues, that achieved durable complete remission after paclitaxel–carboplatin plus bevacizumab chemotherapy followed by maintenance therapy with bevacizumab plus olaparib. This notably occurred in the setting of HRD positivity (GIS 85) without BRCA1/2 mutations. This case highlights the potential role of HRD-directed therapy in gynecologic NEC.

## 2 Case presentation

**Fig. 1:**
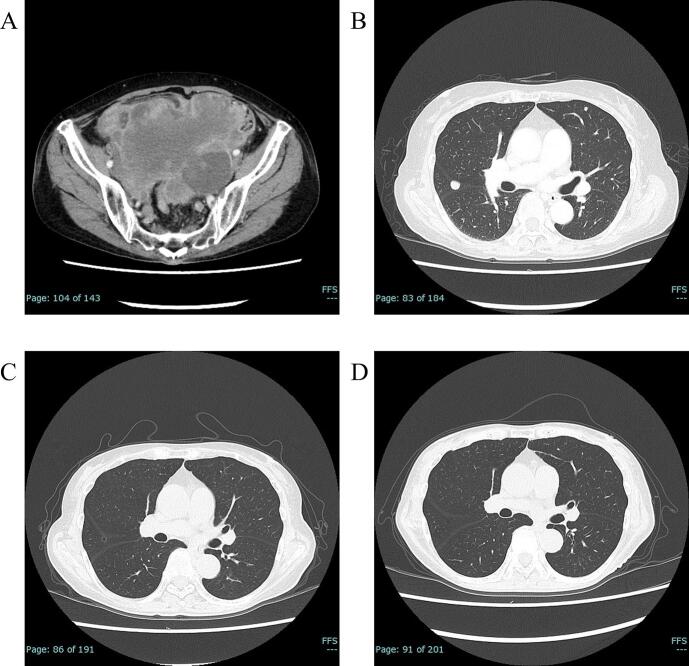
Contrast-enhanced computed tomography findings. (A) Heterogeneously enhancing irregular pelvic mass. (B) Multiple pulmonary nodules consistent with metastatic disease. (C) Partial response achieved after chemotherapy. (D) Sustained complete response during maintenance therapy.

A 71-year-old gravida 2, para 2 woman with no significant medical history presented to a local hospital with a complaint of a lower abdominal mass. Contrast-enhanced computed tomography (CT) revealed a pelvic mass measuring approximately 10 cm, prompting referral to our institution for further evaluation and treatment. Serum tumor markers were markedly elevated, namely lactate dehydrogenase (LDH) at 1875 U/L, cancer antigen 125 (CA125) at 711 U/mL, carcinoembryonic antigen at 91 ng/mL, carbohydrate antigen 19–9 (CA19-9) at 141 U/mL, and human epididymis protein 4 (HE4) at 120 pmol/L. Subsequent chest imaging revealed multiple pulmonary nodules (Fig. 1A, B); the initial clinical diagnosis was advanced ovarian cancer with pulmonary metastases.

Primary debulking surgery was selected despite the presence of pulmonary metastases for several reasons: the pelvic tumor was large and symptomatic, a definitive histologic diagnosis and primary site could not be established preoperatively, no dominant pulmonary lesion suggested a lung primary, and the disease was initially managed according to an ovarian cancer-based strategy in which cytoreductive surgery may be considered when technically feasible. The decision was made after multidisciplinary consultation, with postoperative systemic therapy planned. Intraoperatively, the dominant tumor occupied the pelvis between the uterus and the rectosigmoid colon. The bilateral adnexa could not be identified separately from the tumor. Invasion of the posterior wall of the uterine corpus was suspected, whereas the uterine cervix appeared intact. Multiple peritoneal disseminated lesions were observed throughout the abdominal cavity. Total hysterectomy, bilateral salpingo-oophorectomy, omentectomy, pelvic peritonectomy, sigmoid colon-to-rectal resection, and colostomy were performed. Complete macroscopic resection of the pelvic tumor was achieved; however, residual disseminated intra-abdominal disease exceeding 2 cm remained. The multiple small pulmonary nodules were not surgically resected.

**Fig. 2:**
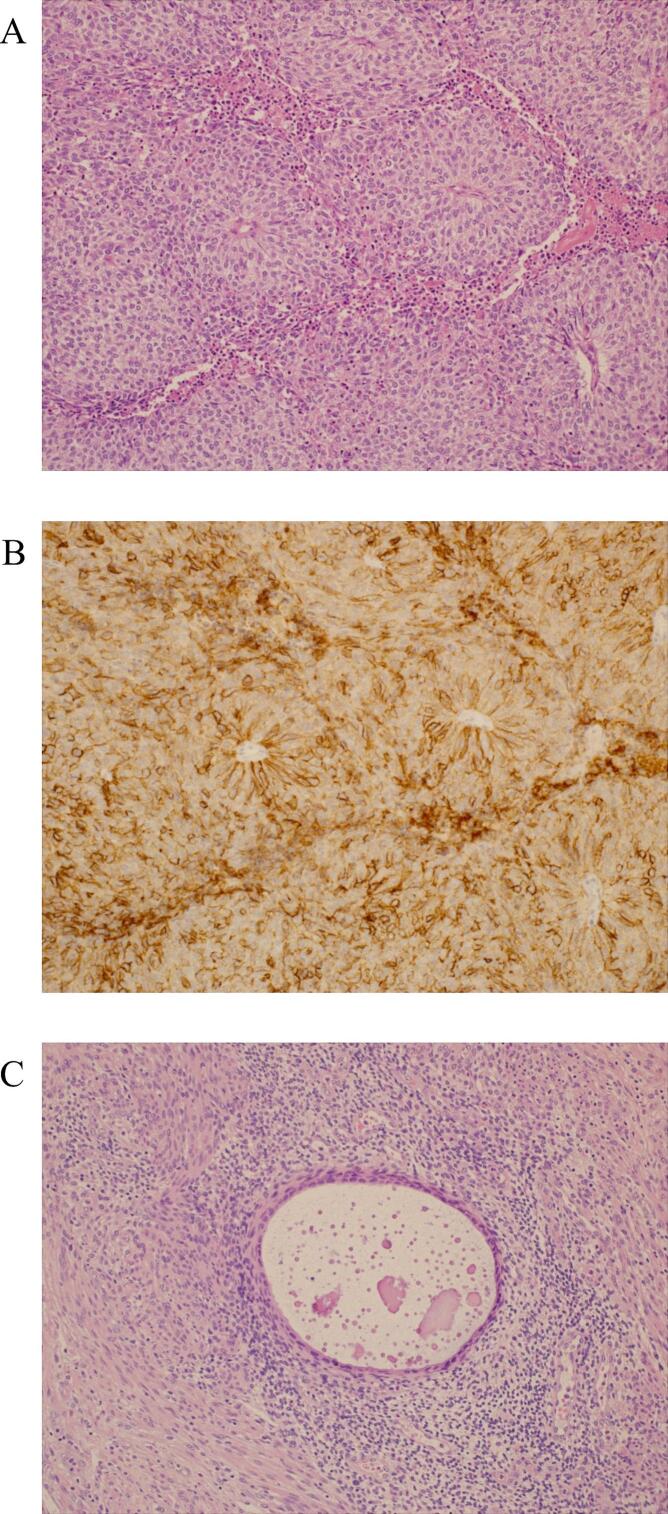
Histological and immunohistochemical findings. (A) Hematoxylin and eosin staining showing sheets of atypical cells with rosette-like structures. (B) Tumor cells demonstrating diffuse CD56 positivity. (C) Endometriotic lesion composed of endometrial glands and stroma with inflammatory cell infiltration.

Histopathological examination revealed LCNEC (Fig. 2A-C). Pulmonary NEC was initially considered based on epidemiologic frequency, but chest imaging revealed multiple small pulmonary nodules consistent with metastatic disease rather than a dominant primary lesion. Accordingly, this case was deemed to have a pelvic primary site with lung metastases. No neoplastic lesions were identified in the uterine cervix or endometrium. Adenomyosis was present in the myometrium, and endometriosis was observed in the uterine serosa. Normal left adnexal structures could not be identified histologically, suggesting complete replacement by tumor tissue. The potential primary sites included Müllerian-derived tissues, such as the left ovary, fallopian tube, adenomyosis, or endometriosis-associated tissue, although a specific definitive origin could not be determined.

Postoperatively, the pathology, imaging findings, and treatment strategy were reviewed in a multidisciplinary setting, and paclitaxel and carboplatin plus bevacizumab were administered based on an ovarian cancer regimen. During treatment, HRD testing using myChoice CDx demonstrated no pathogenic BRCA1/2 variants, but there was a markedly elevated genomic instability score (GIS 85) consistent with HRD. Serum tumor markers were normalized during chemotherapy and remained within the normal range thereafter. After six cycles of chemotherapy, CT revealed shrinkage of pulmonary lesions and no progression of intra-abdominal disease, consistent with a partial response according to RECIST criteria (Fig. 1C). The measurable residual lesions after chemotherapy were therefore the pulmonary metastases. Maintenance therapy with bevacizumab plus olaparib was subsequently initiated. Five months later, follow-up CT demonstrated complete radiologic disappearance of all pulmonary metastases (Fig. 1D). The patient remained disease-free on imaging one year after initiating maintenance therapy.

## 3 Discussion

Gynecologic large-cell neuroendocrine carcinoma is associated with an extremely poor prognosis. In a population-based analysis using surveillance, epidemiology, and end-results data, ovarian and endometrial LCNEC had a median overall survival of 11 months and 5-year overall survival rates below 20%, underscoring the aggressive clinical course and limited effectiveness of conventional treatments, particularly in advanced-stage disease (Pang et al., 2022). Given this context, this case illustrates the emerging role of HRD-directed therapy, including PARP inhibitor–based maintenance, in gynecologic NEC.

Because gynecologic NECs are rare and highly aggressive, systemic treatment has largely been extrapolated from SCLC, with platinum-based chemotherapy forming the backbone of treatment (Stumpo et al., 2023). Cervical NEC has relatively well-established guideline-based multimodal strategies that incorporate platinum and etoposide (Tempfer et al., 2018). On the other hand, for non-cervical gynecologic NECs, including ovarian NEC, the evidence remains limited to retrospective studies and case reports (Stumpo et al., 2023). Consequently, there is still no standard regimen, although etoposide plus platinum and paclitaxel plus carboplatin are commonly used (He et al., 2024, Stumpo et al., 2023).

In the present case, the tumor most likely arose from Müllerian-derived tissues, such as the left ovary, fallopian tube, adenomyosis, or endometriosis-associated tissue. Accordingly, paclitaxel plus carboplatin was selected as the initial regimen, consistent with treatment strategies for epithelial ovarian cancer. Given the limited efficacy of conventional chemotherapy in LCNEC and its poor prognosis (He et al., 2024), we adopted a treatment strategy informed by the PAOLA-1 trial. Although the evidence is not disease-specific, the PAOLA-1 trial demonstrated the clinical benefit of platinum-based chemotherapy with bevacizumab followed by maintenance therapy with bevacizumab plus olaparib in advanced ovarian cancer, which was then extrapolated to this rare setting (Ray-Coquard et al., 2019).

Evidence supporting bevacizumab in NEC remains limited. Some studies suggest its potential activity of bevacizumab-containing regimens in high-grade gastrointestinal or gastroenteropancreatic NECs, but randomized data have not demonstrated a clear survival benefit (Alifieris et al., 2020, Walter et al., 2023). Its role should therefore be considered exploratory. Although the contribution of bevacizumab cannot be definitively determined, its use in the present case was clinically reasonable considering its established role in ovarian cancer, potentially contributing to the observed response.

The use of PARP inhibitors is well-established in ovarian cancer, where their efficacy is closely linked to HRD, particularly in BRCA1/2-mutated tumors. Notably, HRD serves as a validated biomarker for treatment selection (Ray-Coquard et al., 2019). Meanwhile, the development of PARP inhibitors in SCLC is driven by a broader biological rationale. SCLC is characterized by high replication stress, genomic instability, and increased PARP1 expression, suggesting a dependence on DNA damage response pathways (Pratama et al., 2024). Although SCLC and LCNEC are both classified as poorly differentiated high-grade neuroendocrine carcinomas, their molecular features are not identical. SCLC is relatively uniformly characterized by frequent TP53 and RB1 inactivation, whereas LCNEC is molecularly heterogeneous and may include both SCLC-like and non-small-cell carcinoma–like molecular subtypes. Data regarding the frequency of HRD positivity in LCNEC, particularly gynecologic LCNEC, remain extremely limited, and HRD has not been established as a validated biomarker in this disease. Clinical trials have demonstrated that PARP inhibitor-containing regimens can improve progression-free survival and objective response rates, although the overall survival benefit remains limited (Pratama et al., 2024). This biological rationale has been extended to non-pulmonary NECs. Although prospective evidence is lacking, emerging evidence suggests that a subset of NEC harbors alterations in the DNA repair pathway (Misawa et al., 2025, Rose and Sierk, 2019, Wang et al., 2024). Furthermore, cases of cervical NEC with BRCA2 alterations have achieved prolonged disease control with PARP inhibitors (Rose and Sierk, 2019). Therefore, HRD-related mechanisms may be clinically relevant in selected NECs, but the markedly elevated GIS observed in the present case should be interpreted as a hypothesis-generating finding rather than evidence supporting a standard treatment approach.

The present case further supports the potential role of PARP inhibitor–based therapy in gynecologic NEC. The observed clinical response is consistent with the established HRD-driven paradigm in ovarian cancer and the emerging evidence regarding DNA repair vulnerability in NEC. This case highlights the importance of molecular profiling and suggests that PARP inhibitors can be a promising therapeutic option for selected patients with NEC.

## 4 Conclusion

This report describes a rare case of HRD-positive LCNEC of presumed gynecologic origin with multiple pulmonary metastases that achieved durable complete remission on maintenance therapy with bevacizumab plus olaparib after initial platinum-based chemotherapy. HRD assessment, even in the absence of BRCA mutations, may identify a therapeutically actionable subset of NEC beyond the conventional indications of epithelial ovarian cancer.

## 5 Patient consent for publication

Written informed consent was obtained from the patient for the publication of this case report and accompanying images. A copy of the written consent form is available for review by the Editor-in-Chief of this journal on request.

## 6 Declaration of Generative AI Use

Generative AI (ChatGPT, OpenAI; accessed December 2025) was used only for language editing and clarity. The authors reviewed and verified all content and take full responsibility for the manuscript.

## CRediT authorship contribution statement

**Yasushi Iida:** Writing – original draft, Visualization, Investigation. **Yoko Saito:** Writing – review & editing, Investigation. **Miwako Shimazaki:** Writing – review & editing. **Teppei Ichikawa:** Writing – review & editing. **Taichi Irie:** Writing – review & editing. **Jun Matsushima:** Writing – review & editing, Visualization. **Makoto Iizuka:** Writing – review & editing. **Satoshi Takakura:** Writing – review & editing, Supervision.

## Funding

No funding was received.

## Declaration of competing interest

The authors declare that they have no known competing financial interests or personal relationships that could have appeared to influence the work reported in this paper.

## References

[b0005] Alifieris C.E., Griniatsos J., Delis S.G., Nikolaou M., Avgoustou C., Panagiotidis M.I. (2020). Capecitabine, Oxaliplatin, Irinotecan, and Bevacizumab Combination Followed by Pazopanib Plus Capecitabine Maintenance for High-Grade Gastrointestinal Neuroendocrine Carcinomas. Am. J. Clin. Oncol..

[b0010] Dasari A., Mehta K., Byers L.A., Sorbye H., Yao J.C. (2018). Comparative study of lung and extrapulmonary poorly differentiated neuroendocrine carcinomas: a SEER database analysis of 162,983 cases. Cancer.

[b0015] He Q., Wang C., Huang D., Shen J., Liu R., Guan Y. (2024). Clinicopathologic feature and treatment progress of high-grade ovarian neuroendocrine tumors. Med. Oncol..

[b0020] Misawa A., Shingo M., Miyamura T., Ogawa T., Morioka M. (2025). Small Cell Carcinoma of the Ovary, Pulmonary Type, with a Germline BRCA2 Mutation: a Report of a Rare Case. Cureus.

[b0025] Pang L., Chen J., Chang X. (2022). Large-cell neuroendocrine carcinoma of the gynecologic tract: Prevalence, survival outcomes, and associated factors. Front. Oncol..

[b0030] Pratama S., Wiyono L., Setiawan M.S., Lauren B.C. (2024). PARP inhibitors as therapy for small cell lung carcinoma: a systematic review and meta-analysis of clinical trials. Cancer Treat Res Commun..

[b0035] Ray-Coquard I., Pautier P., Pignata S., Pérol D., González-Martín A., Berger R. (2019). Olaparib plus Bevacizumab as First-Line Maintenance in Ovarian Cancer. N. Engl. J. Med..

[b0040] Rindi G., Mete O., Uccella S., Basturk O., La Rosa S., Brosens L.A.A. (2022). Overview of the 2022 WHO Classification of Neuroendocrine Neoplasms. Endocr. Pathol..

[b0045] Rose P.G., Sierk A. (2019). Treatment of neuroendocrine carcinoma of the cervix with a PARP inhibitor based on next generation sequencing. Gynecol Oncol Rep..

[b0050] Stumpo S., Formelli M.G., Persano I., Parlagreco E., Lauricella E., Rodriquenz M.G. (2023). Extrapulmonary Neuroendocrine Carcinomas: Current Management and Future Perspectives. J. Clin. Med..

[b0055] Tempfer C.B., Tischoff I., Dogan A., Hilal Z., Schultheis B., Kern P. (2018). Neuroendocrine carcinoma of the cervix: a systematic review of the literature. BMC Cancer.

[b0060] Walter T., Lievre A., Coriat R., Malka D., Elhajbi F., Di Fiore F. (2023). Bevacizumab plus FOLFIRI after failure of platinum-etoposide first-line chemotherapy in patients with advanced neuroendocrine carcinoma (PRODIGE 41-BEVANEC): a randomised, multicentre, non-comparative, open-label, phase 2 trial. Lancet Oncol..

[b0065] Wang M., Gao F., Wang X., Guo Y., Zhang H. (2024). Pure large-cell neuroendocrine carcinoma of the ovary with a somatic BRCA1 mutation: the first reported case and the review of the literature. SAGE Open Med. Case Rep..

[b0070] Yachida N., Yoshihara K., Yamaguchi M., Suda K., Tamura R., Enomoto T. (2021). How does Endometriosis Lead to Ovarian Cancer? the Molecular Mechanism of Endometriosis-Associated Ovarian Cancer Development. Cancers (Basel).

[b0075] Yanagita T., Hikichi T., Waragai Y., Shimizu H., Takahashi Y., Abe N. (2023). Mixed high-grade serous and large cell neuroendocrine carcinoma arising from rectal endometriosis 11 years after hysterectomy. Clin. J. Gastroenterol..

